# Molecular surveillance of *Plasmodium vivax* drug resistance genes in Kanchanaburi province, Thailand, from 2022 to 2025

**DOI:** 10.1186/s12936-026-06099-1

**Published:** 2026-08-07

**Authors:** Kanyarat Boonpeng, Phiraya Pitchayatanakorn, Kaewkanha Kijprasong, Suttipat Srisutham

**Affiliations:** 1 Department of Clinical Microscopy, Faculty of Allied Health Sciences, Chulalongkorn University, Bangkok, 10330 Thailand; 2 Phayabalsathanphrabaramee Hospital, Naung Proe, Kanchanaburi, Thailand

**Keywords:** *Plasmodium vivax*, *Pvmdr1*, *Pvdhfr*, *Pvdhps*, *Pvcrt-o*

## Abstract

**Background:**

*Plasmodium vivax* remains prevalent in Thailand’s border regions, including Kanchanaburi, where concerns about antimalarial drug resistance persist. However, data on molecular markers associated with drug resistance in this area remain limited. This study aimed to describe the distribution of mutations in four putative *P. vivax* drug resistance genes in isolates from this region.

**Methods:**

Blood samples (n = 100) were collected from patients with confirmed *P. vivax* infections in Kanchanaburi, Thailand, between 2022 and 2025. Genomic DNA was analyzed using nested PCR and Sanger sequencing to examine four putative drug resistance genes (*Pvmdr1, Pvdhfr, Pvdhps,* and *Pvcrt-o*).

**Results:**

The *Pvmdr1* Y976F mutation was detected in 7.07% of samples, with the **M**_**958**_Y_976_**L**_**1076**_ haplotype being the most common (50.51%). The *Pvcrt-o* K10 insertion and K32T substitution were observed in 31.17% and 38.96% of isolates, respectively. The quadruple-mutant *Pvdhfr* haplotype (**I**_**57**_**R**_**58**_**M**_**61**_**T**_**117**_) was found in 42.42%, while the wild-type *Pvdhps* allele was present in 49.98%. Nineteen distinct multi-locus genotype profiles were identified, the most prevalent being **M**_**958**_Y_976_**L**_**1076**_–**L**_**57**_S_58_T_61_S_117_–A_383_K_512_A_553_–without K10 insertion/**T**_**32**_ (36.49%), followed by **M**_**958**_Y_976_**L**_**1076**_–**F**_**57**_**S**_**58**_**T**_**61**_**S**_**117**_–A_383_K_512_A_553_–with **K10 insertion/**T_32_ (12.16%).

**Conclusions:**

This study provides the descriptive analysis of genetic diversity and multi-locus combinations of resistance-associated markers across four putative drug-resistance genes in *P. vivax* isolates from Kanchanaburi, Thailand, and offers updated data to inform and support malaria treatment strategies in this region.​

**Supplementary Information:**

The online version contains supplementary material available at https://doi.org/10.1186/s12936-026-06099-1.

## Background

Malaria continues to pose a major threat to global public health. In 2024, the World Health Organization (WHO) reported an estimated 282 million malaria cases and 610,000 related deaths worldwide [1]. Although the WHO set targets for Thailand to reduce malaria incidence by 25–70% by 2025, surveillance data indicate a 46.4% increase in malaria cases between 2022 and 2023 [2]. Notably, rising malaria incidence in Myanmar has also resulted in cross-border transmission into western Thailand, posing a risk to progress achieved in the subregion [1]. *Plasmodium vivax* is one of the most important malaria species and is the predominant human malaria parasite, particularly in Southeast Asia (SEA), where 52.4% of malaria cases were attributed to *P. vivax* [1]. *P. vivax* is more difficult to eliminate because asymptomatic infections and latent hypnozoites often go undiagnosed and untreated [3]. Consequently, these factors underscore the growing importance of *P. vivax* as a public health concern.

Thailand’s national antimalarial drug policy, the standard treatment for *P. vivax* malaria consists of a three-day chloroquine (CQ) regimen combined with a 14 day course of primaquine for radical cure [4]. Kanchanaburi Province is one of Thailand’s malaria-endemic provinces bordering Myanmar and has consistently ranked high for antimalarial drug resistance [5]. With transmission persisting despite intensive control efforts, high levels of population mobility in this region contribute substantially to cross-border malaria transmission along the Thai–Myanmar border [6]. Evidence indicates a high prevalence of CQ resistance–associated mutations in this region [7, 8]. Regular monitoring of CQ resistance is needed to detect further development of parasite resistance in this area [7].

Molecular markers of antimalarial resistance, linked to decreased parasite sensitivity to antimalarial agents, have become essential tools for the detection of resistance, surveillance of emerging trends, and the development of evidence-based response strategies [9]. Although CQ has been used effectively for the treatment of *P. vivax* in many SEA countries [10–13], CQ resistance in *P. vivax* has been reported in Myanmar [11, 13], Vietnam [14], including Thailand [8, 15–18]. The potential molecular markers most commonly investigated for CQ resistance are the multidrug resistance 1 gene (*Pvmdr1*) and the chloroquine resistance transporter orthologue gene (*Pvcrt-o*) [17, 19]. *Pvmdr1* Y976F mutation and the *Pvcrt-o* lysine insertion at codon 10 (K10 insertion) are associated with reduced CQ susceptibility [15, 17]. In addition, increased *Pvcrt-o* gene copy number has been linked to a CQ-resistant phenotype [20]. The *P. vivax* dihydrofolate reductase (*Pvdhfr*) and dihydropteroate synthase (*Pvdhps*) genes, which are associated with resistance to sulfadoxine–pyrimethamine (SP), became clinically relevant following the introduction of SP in Thailand in 1967 for the treatment of mixed *P. falciparum* infections. Notably, resistance to SP was reported in the same year [30]. Mutations in *Pvdhfr* at amino acid positions 57, 58, 61, and 117, as well as in *Pvdhps* at positions 383 and 553, continue to be observed at high prevalence in the border region of Thailand [21, 22]. Despite these concerns, recent data on the current status of *P. vivax* drug resistance–associated markers in Kanchanaburi Province remain limited, underscoring the need for updated molecular surveillance to inform evidence-based malaria treatment strategies. Therefore, we designed this study as a descriptive molecular surveillance of resistance-associated markers in *P. vivax* in this setting.

The present study aimed to describe the prevalence of mutations, haplotype diversity, and multi-locus combinations of resistance–associated markers in four candidate drug resistance genes (*Pvmdr1*, *Pvdhfr*, *Pvdhps*, and *Pvcrt-o*) in *P. vivax* isolates from Kanchanaburi Province, Thailand.

## Methods

### Study design and sample collection

This study was a descriptive molecular surveillance of *P. vivax* conducted at Kanchanaburi, Thailand from 2022 to 2025. One hundred confirmed *P. vivax*–positive samples were analyzed according to their availability, without formal inferential sample size calculation. Symptomatic malaria patients were recruited through passive case detection. Specimen collection periods for *P. vivax*–infected patients are described in Table 1. Venous EDTA whole blood was collected from each patient and stored at 4 °C until DNA extraction, after which the extracted DNA was stored at − 20 °C until further analysis.

**Table 1:** Specimen collection periods for *P. vivax*–infected patients

Year	Collection period	Number of *P. vivax* specimens
2022	January–May	25
2023	June–November	25
2024	March–December	25
2025	March–May	25
Total		100

### DNA extraction and molecular confirmation of *Plasmodium* spp.

Genomic DNA was isolated from 200 µL of EDTA-blood samples using the QIAamp DNA Blood Kit (Qiagen, Germany), following the manufacturer’s protocol. DNA was eluted in 200 µL of the provided elution buffer and subsequently stored at − 20 °C until further analysis. Molecular confirmation of the *Plasmodium* species was performed using conventional nested polymerase chain reaction (PCR) with species-specific primers, as detailed in Supplementary Table S1. Only *P. vivax* monoinfections confirmed by the PCR were included. Nested PCR was performed using a T100 Thermal Cycler (Bio-Rad Laboratories, Inc., USA). For the first round (nest-1), the rPLU-1 and rPLU-5 primer pair was used to amplify four *Plasmodium* species, whereas for *P. knowlesi* detection, the rPLU-5 and rPLU-6 primer pair was employed. The thermal cycling conditions consisted of an initial denaturation at 94 °C for 5 min, followed by 25 cycles of denaturation at 94 °C for 30 s, annealing at 58 °C for 30 s, and extension at 72 °C for 45 s, with a final extension at 72 °C for 7 min. The second round (nest-2) was performed under similar conditions, except that the annealing temperature was adjusted to 64 °C for 30 s and the number of cycles was increased to 30. For *P. knowlesi* detection in nest 2, the annealing temperature was set at 55 °C for 30 s. PCR products were verified by electrophoresis on a 3% agarose gel (SERVA Electrophoresis GmbH, Germany).

### Amplification of *P. vivax* drug resistance genes and sequence alignment

Targeted regions of the *P. vivax* drug resistance genes were amplified using nested PCR. Primers and PCR conditions were described in Table 2. The PCR products of each gene were separated by 1.5% agarose gel electrophoresis (SERVA Electrophoresis GmbH, Germany), stained with ViSafe Green (Vivantis Technologies, Selangor, Malaysia), and purified using the PureDirex kit (Bio-Helix, Taiwan). The purified products were then then subjected to Sanger DNA sequencing (Macrogen, Korea). To confirm the identity of the amplified genes, the obtained sequences were compared with GenBank reference sequences using the nucleotide BLAST (BLASTn) search tool (https://blast.ncbi.nlm.nih.gov/Blast.cgi). To identify polymorphisms, the entirety of the sequenced amplicons for the four *P. vivax* drug resistance genes, together with corresponding reference sequences from GenBank, were aligned and analyzed using MEGA X software version 12.1.2 with the ClustalW algorithm [23]. These alignments were rigorously evaluated to screen for both established resistance-associated codons and any potential novel non-synonymous mutations within the extended amplified regions. The reference sequences were obtained from GenBank under accession numbers XM_001613678 (*Pvmdr1*), XM_001615032 (*Pvdhfr*), XM_001617159 (*Pvdhps*), and AF314649.1 (*Pvcrt-o*).

**Table 2:** Primers and PCR conditions used for amplification of *P. vivax* drug resistance marker genes

Target	PCR cycles	Primer sequence (5’-3’)	Product size (bp)	condition	Refs.
*Pvmdr1*	Nest-1	F—CACCGCACCAGTTGATTCCT	2804	94 C, 5 min [94 C 30 s, 62 C 30 s, 72 C 3 min] × 25 cycles, 72 C, 7 min	[8]
		R—CTTATATACGCCGTCCTGCAC			
	Nest-2	F—GGATAGTCATGCCCCAGGATTG	604	94 C, 2 min [94 C 30 s, 62 C 30 s, 72 C 30 s] × 30 cycles, 72 C, 7 min	
		R—CATCAACTTCCCGGCGTAGC			
*Pvdhfr*	Nest-1	F—CACCGCACCAGTTGATTCCT	983	94 C, 5 min [94 C 30 s, 56 C 30 s, 72 C 1 min] × 20 cycles, 72 C, 7 min	
		R—CCTCGGCGTTGTTCTTCT			
	Nest-2	F—CCCCACCACATAACGAAG	755	94 C, 2 min [94 C 30 s, 56 C 30 s, 72 C 30 s] × 25 cycles, 72 C, 7 min	
		R—CCCCACCTTGCTGTAAACC			
*Pvdhps*	Nest-1	F—GATGGCGGTTTATTTGTCG	1009	94 C, 5 min [94 C 30 s, 56˚C 30 s, 72 C 1 min] × 20 cycles, 72 C, 7 min	
		R—GCTGATCTTTGTCTTGACG			
	Nest-2	F—GCTGTGGAGAGGATGTTC	731	94 C, 2 min [94 C 30 s, 56 C 30 s, 72 C 30 s] × 25 cycles, 72 C, 7 min	
		R—CCGCTCATCAGTCTGCAC			
*Pvcrt-o*		F—AAGAGCCGTCTAGCCATCC	1186	94 C, 10 min [94 C 50 s, 61 C 1 min, 72 C 1.5 min] × 35 cycles, 72 C, 7 min	[15]
		R—AGTTTCCCTCTACACCCG			

### Haplotypes analysis of *P. vivax* drug resistance genes

Comparison of drug resistance (*Pvmdr1*, *Pvdhfr*, and *Pvdhps, and Pvcrt*-o) gene diversity among *P. vivax* isolates from Kanchaburi province, Thailand. DnaSP Version 6.12.03 was used to estimate the number of haplotypes (H), haplotype diversity (Hd) and nucleotide diversity (π). *P. vivax* drug resistance genes were combined for each sample to determine the prevalence of common haplotypes.

### Molecular modeling

The putative peptide models of the wild-type *Pv*CRT-o transporter and the *Pv*CRT-o with the novel K32T mutant were predicted using AlphaFold2 [24]. Peptide structures were visualized by using PyMOL (The PyMOL Molecular Graphics System, Version 3.0 Schrödinger, LLC.)

### Statistical analysis

Statistical analyses were performed using IBM SPSS Statistics (version 29.0.2.0) and visualized using Prism software (GraphPad Inc., San Diego, USA).

## Results

### Prevalence of individual mutation in *Pvmdr1*, *Pvdhfr*, *Pvdhps*, and *Pvcrt-o *in *P. vivax* in Kanchanaburi province, Thailand

**Fig. 1:**
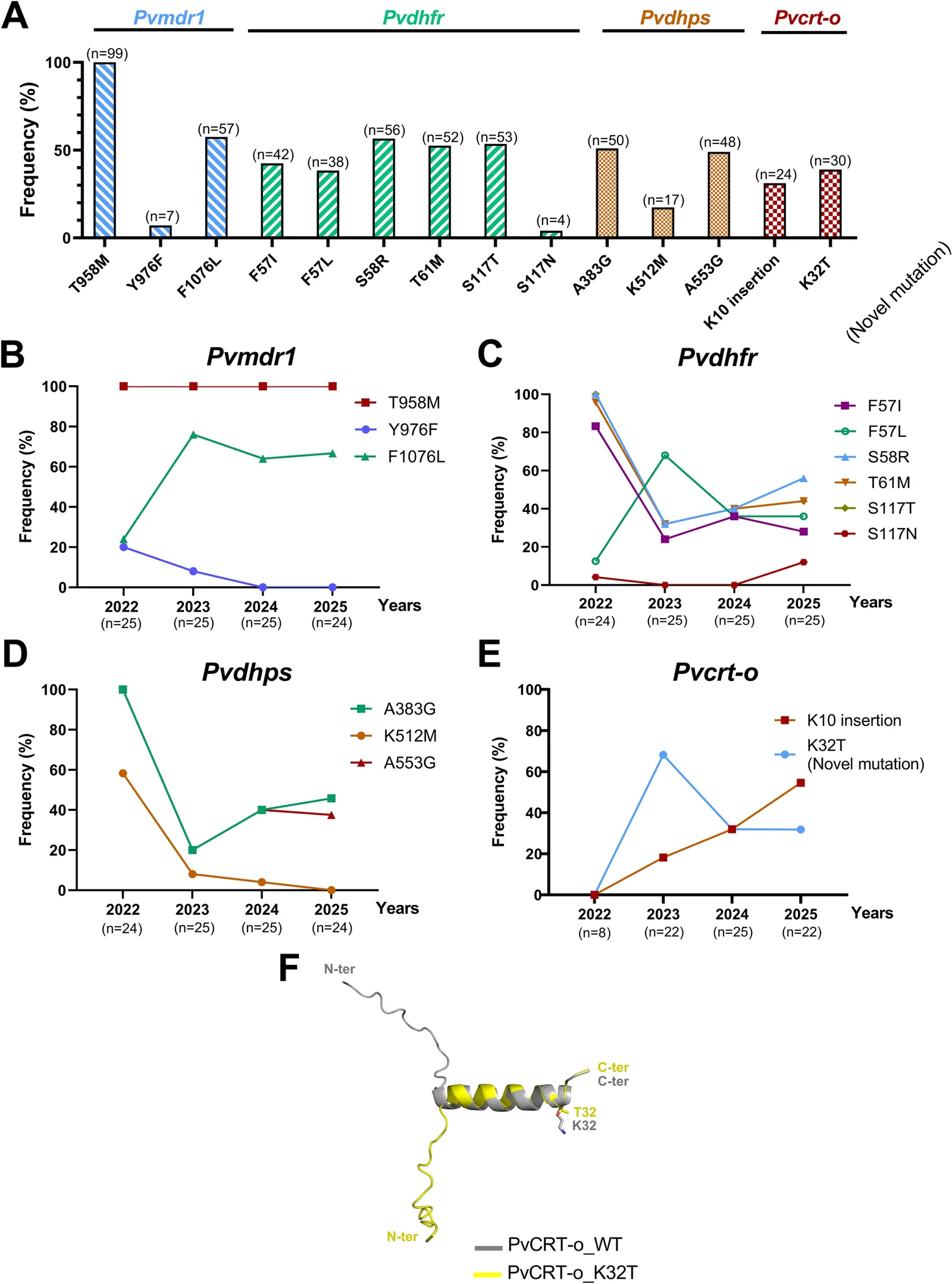
Prevalence of drug resistance–associated mutations in *P. vivax* isolates, including a *Pvcrt-o* K32T mutation. **A** Prevalence of individual mutations in *Pvmdr1*, *Pvdhfr*, *Pvdhps*, and *Pvcrt-o* among *P. vivax* isolates. **B–E** Annual prevalence of mutations in *Pvmdr1*, *Pvdhfr*, *Pvdhps*, and *Pvcrt-o* from 2022 to 2025, respectively. **F** Putative peptide structural models of PvCRT-o variants generated using AlphaFold2 and visualized in PyMOL. Superimposition of the wild-type peptide (gray) and the K32T mutant (yellow), with residues K32 and T32 shown as sticks

The ***Pvmdr1*** gene was successfully amplified and sequenced in 99% (99/100) of isolates. Mutations at *Pvmdr1* codon 958 were observed in all successfully sequenced *P. vivax* isolates (99/99, 100%), while the Y976F and F1076L mutations were detected at frequencies of 7.07% (7/99) and 57.58% (57/99), respectively (Fig. 1A, blue bars). The ***Pvdhfr*** gene was successfully sequenced in 99% of samples (99/100). Mutations at codons F57I, F57L, S58R, T61M, S117T, and S117N were observed in 42.42% (42/99), 38.38% (38/99), 56.57% (56/99), 52.53% (52/99), 53.54% (53/99), and 4.04% (4/99) of isolates, respectively (Fig. 1A, green bars). The ***Pvdhps*** gene was successfully sequenced in 98% of samples (98/100). Mutations at codons A383G, K512M, and A553G were detected at frequencies of 51.02% (50/98), 17.35% (17/98), and 48.98% (48/98), respectively (Fig. 1A, orange bars). The ***Pvcrt-o*** gene was successfully sequenced in 77% of isolates (77/100). The K10 insertion was detected in 31.17% (24/77) of isolates, and a novel K32T mutation was identified in 38.96% (30/77) of isolates (Fig. 1A, red bars*)* (Fig. 1A, red bars). The detailed sequencing success status and specific mutations detected for all four genes per individual isolate are summarized in Supplementary Table S2. The corresponding raw nucleotide sequence data in FASTA format are provided in Supplementary Table S3. Across the four-year study period (2022–2025), the ***Pvmdr1*** T958M mutation was detected at a frequency of 100% in all years. In contrast, the Y976F mutation, previously associated with chloroquine resistance [15, 17], was detected in 20% and 8% of isolates in 2022 and 2023, respectively, and was not detected in *P. vivax* isolates collected in 2024–2025 (Fig. 1B). The F1076L mutation was observed in 76% of isolates in 2023 (76%) and approximately 60% of isolates in 2024–2025 (Fig. 1B). For ***Pvdhfr***, mutations at positions F57I, S58R, T61M, and S117T were observed at frequencies around 40% in 2023 and between approximately 20–60% in 2025, whereas the S117N mutation was observed at approximately 0–4% (Fig. 1C). In ***Pvdhps***, mutations at positions A383G, K512M, and A553G were observed at frequencies of around 20% in some years and approximately 40% in 2025 (Fig. 1D). In ***Pvcrt-o***, the K10 insertion was not detected in 2022 but was observed at a frequency of 54.55% in 2025, while the novel K32T mutation was observed at 68% in 2023 and at approximately 30% in 2024–2025 (Fig. 1E).

The novel mutation was identified as a nucleotide substitution (c.32 AAG > ACG) (Supplementary Fig. S4), and the corresponding putative peptide structural model is shown in Fig. 1F. In this predictive model, a localized alteration near the N-terminal region is observed. The threonine substitution at codon 32 (T32), depicted as yellow sticks (Fig. 1F), may be associated with a subtle change in the orientation of the adjacent helical segment compared with the wild-type lysine (K32), although the overall helical structure appears conserved. These structural observations should be regarded as descriptive and hypothesis-generating only, as there is currently no functional or clinical evidence that K32T alters chloroquine interaction or resistance.

### Haplotypes analysis of *Pvmdr1*, *Pvdhfr*, *Pvdhps*, and *Pvcrt-o *in *P. vivax* in Kanchanaburi province, Thailand

**Fig. 2:**
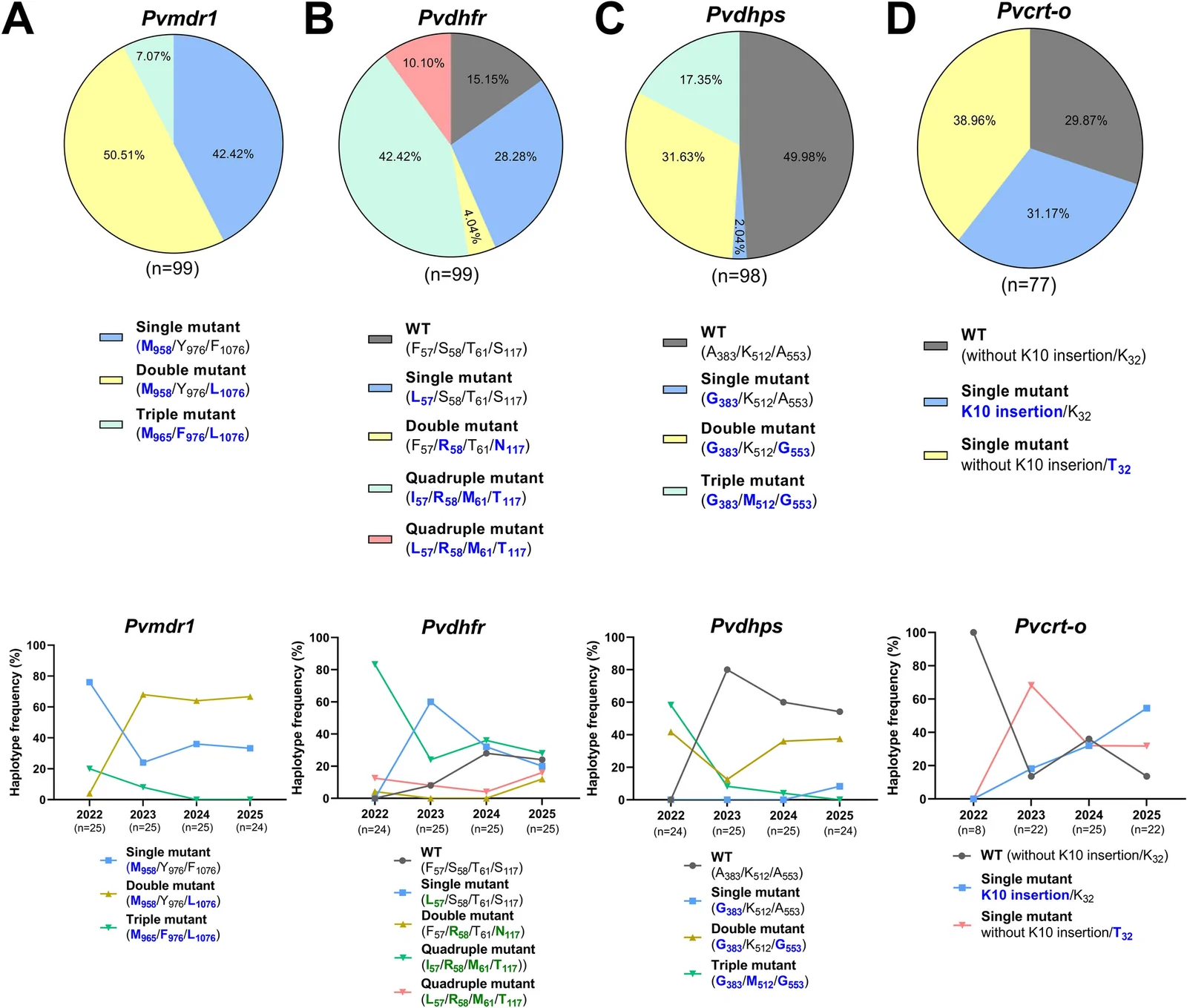
Haplotype analysis of *P. vivax* isolates from Kanchanaburi Province, Thailand. **A** Prevalence of *Pvmdr1*, *Pvdhfr*, *Pvdhps*, and *Pvcrt-o* haplotypes among *P. vivax* isolates from Kanchanaburi Province, Thailand. Pie charts show haplotype frequencies expressed as percentages among 100 *P. vivax* isolates, with mutant amino acids indicated in bold blue text. **B** Year-by-year distribution of *Pvmdr1*, *Pvdhfr*, *Pvdhps*, and *Pvcrt-o* haplotypes from 2022 to 2025; the x-axis represents time in years

*Pvmdr1* haplotypes comprised three variants: a single-mutant allele (**M**_**958**_ Y_976_F_**1076**_), which was predominant (42.42%); a double-mutant allele (**M**_**958**_Y_976_**L**_**1076**_) (50.51%); and a triple-mutant allele (**M**_**958**_**F**_**976**_**L**_**1076**_), associated with CQ resistance [15, 17], detected in 7.07% of isolates (Fig. 2A, upper panel).Over the study period, the single-mutant haplotype (**M**_**958**_Y_976_F_1076_) was observed less frequently in later years, while the double-mutant haplotype (**M**_**958**_Y_976_**L**_**1076**_) reached frequencies of up to about 70% during 2023–2025. In contrast, the triple-mutant haplotype (**M**_**958**_**F**_**976**_**L**_**1076**_) was not detected in isolates collected in 2024–2025 (Fig. 2A, lower panel). Several distinct Pvdhfr haplotypes were identified based on variations at codons 57, 58, 61, and 117 [22]. Analysis of 5 main haplotypes revealed that the quadruple mutant (**I**_**57**_**R**_**58**_**M**_**61**_**T**_**117**_) predominates (42.42%), followed by single mutant (F_57_**R**_**58**_T_61_S_117_) (28.28%), wild type allele (15.15%), quadruple mutant (**L**_**57**_**R**_**58**_**M**_**61**_**T**_**117**_) (10.10%), and double mutant allele (F_57_**R**_**58**_T_61_**N**_**117**_) (4.04%), respectively (Fig. 2B, upper panel). Notably, the prevalence of the major quadruple-mutant haplotype (**I**_**57**_**R**_**58**_**M**_**61**_**T**_**117**_) by approximately 30% between 2023 and 2025, while the other haplotypes remained at relatively low (Fig. 2B, lower panel). Pvdhps haplotypes were defined by different combinations of mutations at codons 383, 512, and 553 [21, 22]. The four major Pvdhps haplotypes were the wild type (49.98%), followed by the double-mutant allele (**G**_**383**_K_512_**G**_**553**_) (31.63%), triple mutant allele (**G**_**383**_**M**_**512**_**G**_**553**_) (17.35%), and single mutant allele (**G**_**383**_K_512_A_553_) (2.04%), respectively (Fig. 2C, upper panel). The wild-type major haplotype remained predominant, accounting for approximately 60–80% of isolates during 2023–2025 (Fig. 2C, lower panel). Given the limited annual sample sizes, these haplotypes distributions should be interpreted as descriptive, and any potential selection pressure acting on *Pvdhfr* and *Pvdhps* in this setting remains speculative.

Analysis of *Pvcrt-o* haplotypes identified three major haplotypes. The most prevalent was the single-mutant allele without the K10 insertion/**T**_**32**_, accounting for 38.96% of *P. vivax* isolates, followed by the haplotype with the K10 insertion/**K**_**32**_ (31.17%) and the wild-type allele (29.87%) (Fig. 2D, upper panel). Notably, the K10 insertion and K32T mutation in *Pvcrt-o* were not observed together. The mutant allele (K10 insertion with K32 and K10 insertion without K32) were observed in 2023–2025 (Fig. 2D, lower panel).

Haplotype diversities (Hd) and nucleotide diversity (π) for *Pvmdr1*, *Pvdhfr*, *Pvdhps*, and *Pvcrt-o* were calculated to summarize sequence variation, and the detailed values are provided as descriptive supporting information in Supplementary Data 5.

### Descriptive multi-locus marker profiles of *Pvmdr1*, *Pvdhfr*, *Pvdhps*, and *Pvcrt-o *in *P. vivax* from Kanchanaburi province, Thailand

**Fig. 3:**
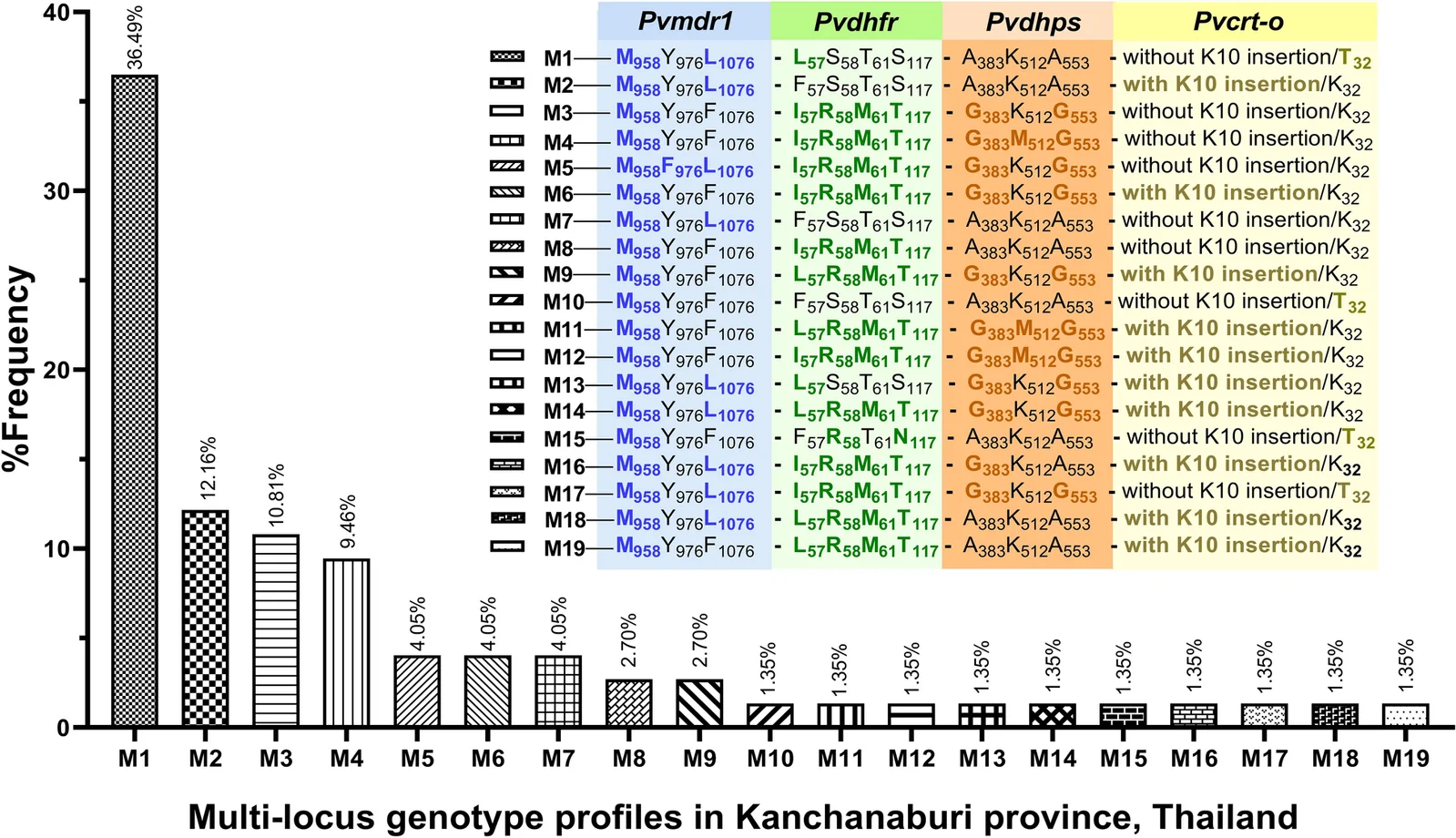
Multi-locus marker profiles of putative drug-resistance genes (*Pvmdr1*, *Pvdhfr*, *Pvdhps*, and *Pvcrt-o*) in Kanchanaburi Province, Thailand. Data are presented as the percentage frequency of isolates (n = 74). M1–M19 represent standard haplotypes 1–19, ordered from highest to lowest frequency. Bold and highlighted letters indicate mutations at each codon position

Of the 100 isolates, complete multi-locus marker profiles across all four target genes (*Pvmdr1*, *Pvdhfr*, *Pvdhps*, and *Pvcrt-o*) were successfully obtained for 74 isolates. By combining the observed alleles at these loci, 19 distinct multi-locus marker combinations (M1–M19) were identified in *P. vivax* isolates from Kanchanaburi Province (Fig. 3A). The most frequently observed profile (M1), **M**_**958**_Y_976_**L**_**1076**_–**L**_**57**_S_58_T_61_S_117_–A_383_K_512_A_553_–without K10 insertion/**T**_**32**_, accounted for 36.49% of isolates and did not include the *Pvmdr1* Y976F mutation, while carrying a single-mutant *Pvdhfr* allele. The second most common profile (M2), **M**_**958**_Y_976_**L**_**1076**_–**F**_**57**_S_58_T_61_S_117_–A_383_K_512_A_553_–**K10 insertion**/K_32_ was observed in 12.16% of isolates. M3 (**M**_**958**_Y_976_F_1076_–**I**_**57**_**R**_**58**_**M**_**61**_**T**_**117**_–**G**_**383**_K_512_**G**_**553**_–without K10 insertion/K_32_, 10.81%) and M4 (**M**_**958**_Y_976_F_1076_–**I**_**57**_**R**_**58**_**M**_**61**_**T**_**117**_–**G**_**383**_**M**_**512**_**G**_**553**_–without K10 insertion/K_32_, 9.46%) were subsequently identified. The remaining 15 profiles (M5–M19) were each observed at frequencies below 5%.

## Discussion

 This study provides updated descriptive molecular surveillance of four candidate drug resistance –associated markers (*Pvmdr1*, *Pvdhfr*, *Pvdhps*, and *Pvcrt-o*) in *P. vivax* from Kanchanaburi province, western Thailand. Molecular markers of *P. vivax* drug resistance are still not well defined and most candidates investigated to date are homologues of *P. falciparum* genes, including *Pvmdr1, Pvdhfr, Pvdhps, and Pvcrt-o.*

Point mutations in the ***Pvmdr1*** and ***Pvcrt-o*** genes, particularly *Pvcrt-o* K10 insertion and *Pvmdr1* Y976F, have been proposed as candidate markers of CQ resistance in *P. vivax* [15, 17, 19, 25–27] and are supported by in vitro data showing increase CQ IC₅₀ associated with these variants [15, 17]. The *Pvmdr1* Y976F mutation has previously been associated with a 1.7-fold increase in chloroquine IC₅₀ [17, 28] and has been reported at 96% of Papuan isolates and in 25% (2007) [17], 95.5% (2008) and 64.3% (2014) of Thai isolates [18]. In contrast, the Y976F mutant allele has been detected in India (0.86–7%) [29, 30] and Myanmar (4.3–13.3%) [15, 31]. Consistent with these observations, the Y976F mutation was identified in 7.07% of total isolates from Kanchanaburi, Thai–Myanmar border, in recent years. These exploratory findings describe the current prevalence of putative chloroquine resistance-associated mutations in this setting without providing definitive evidence of clinical resistance.

For *Pvcrt-o*, several molecular surveillance studies have reported no detection of the K10 insertion in isolates from the Thai–Myanmar and Thai–Cambodia border regions, northeastern Myanmar [18, 32, 33] and central China [41]. The K10 insertion was identified in isolates from Thailand (56.0%) and Myanmar (46.2%) previously in 2008 [15]. In this study, the *Pvcrt-o* K10 insertion allele was observed at a prevalence of 31.17%. Previous studies have reported *Pvcrt-o* K10 insertion prevalences of 17.6% in Ranong Province, Thailand [16],5.6% in India [29], 13.0% in Brazil [34], and 48.3% (2009) and 72.7% (2016) in parts of Myanmar [35]. Based on our descriptive data, the presence of both *Pvmdr1* Y976F and *Pvcrt-o* K10 insertion alleles in this setting describes the current pattern of putative chloroquine resistance–associated markers, but does not provide definitive evidence of emerging chloroquine resistance. Because this study relied solely on molecular surveillance without accompanying clinical efficacy or pharmacokinetic data, these mutational profiles should be interpreted cautiously, and further functional and clinical investigations are required. Additionally, a novel *Pvcrt-o* K32T (AAG → ACG) substitution was identified in a subset of isolates in this study. At present, there is no functional, clinical, or pharmacological evidence linking the K32T mutation to altered chloroquine interaction or resistance and the model should be regarded only and may inform future hypothesis-driven functional studies.

Although sulfadoxine–pyrimethamine (SP) has not been used for the treatment of *P. vivax* infections in Southeast Asian countries [1] SP-associated resistance in *P. vivax* has been reported through mutations of ***Pvdhfr*** and ***Pvdhps*** [36–38]. Double (**R**_**58**_**N**_**117**_) and quadruple (**L**_**57**_**R**_**58**_**M**_**61**_**T**_**117**_) *Pvdhfr* mutations, which have been linked to higher SP treatment failure in Papua [39], and *Pvdhps* mutations at codons 382, 383, 512, and 553, associated with sulfadoxine resistance, have been observed at varying frequencies across multiple regions[22], have been observed at varying frequencies across multiple regions. Mutant alleles at codons 383 and 553 in *Pvdhps* have been reported at 1–2% in *P. vivax* isolates from East Timor [40], Korea [41], Pakistan [42], Iran [43] and at 22% in northeastern Myanmar (2020), 80.77% in Kalabakan, and 65.1% in Thai border regions.In our recent findings, the *Pvdhfr* quadruple mutant (**I**_**57**_**R**_**58**_**M**_**61**_**T**_**117**_) predominated, accounting for 42.42% of haplotypes, while *Pvdhps* haplotypes were mainly wild type (49.98%), followed by the double-mutant allele (**G**_**383**_K_512_**G**_**553**_) at 31.63%. Given the small annual sample sizes, and the likely wide, overlapping confidence intervals, this pattern should be regarded as descriptive only, and any potential reduction in selective pressure from sulfadoxine–pyrimethamine exposure remains speculative. Larger longitudinal studies will be needed to determine whether this apparent decline truly reflects changes in SP-related selective pressure in the region.

Identification of multi-locus marker profiles across multiple regions can be useful for monitoring the descriptive patterns of putative antimalarial drug resistance markers [44–46]. Our descriptive analysis identified 19 distinct multi-locus marker profiles in *P. vivax* isolates from Kanchanaburi Province, Thailand. The most prevalent four-gene combination (M1) consisted of a double-mutant *Pvmdr1* allele (**M**_**958**_Y_976_ **L**_**1076**_), single-mutant *Pvdhfr* (**L**_**57**_S_58_T_61_S_117_) allele, a wild-type *Pvdhps* allele (A_383_K_512_ A_553_), and *Pvcrt-o* with the **T**_**32**_ mutation. In contrast, the profile M2 —characterized by a double-mutant *Pvmdr1* allele (**M**_**958**_Y_976_ **L**_**1076**_), a wild-type *Pvdhfr* allele (F_57_S_58_T_61_S_117_), a wild-type *Pvdhps* allele (A_383_K_512_A_553_), and *Pvcrt-o* with the K10 insertion—was observed in specimens collected in the later years of the study.

These variations describe the observed combinations and frequencies of resistance-associated markers rather than indicating biologically defined genotypes or evolutionary dynamics. The moderate presence and year-to-year fluctuations of the *Pvcrt-o* K10 insertion underscore the importance of continued molecular surveillance to monitor the distribution of these putative markers over time, but any temporal patterns should be interpreted as descriptive only.

This study has several limitations. First, the overall and annual sample sizes were relatively small, especially for isolates with complete multi-gene data, which reduces statistical power and limits our ability to draw robust temporal inferences about changes in mutation frequencies. Therefore, year-to-year differences observed in this dataset should be interpreted as descriptive and exploratory rather than definitive evidence of temporal trends. Second, because the study area was limited to Kanchanaburi Province, Thailand, further investigations across additional geographic regions are required to obtain more representative estimates of drug resistance–associated mutation frequencies.

## Conclusions

This study provides baseline descriptive molecular surveillance data on four putative drug resistance–associated genes (*Pvmdr1, Pvdhfr, Pvdhps,* and *Pvcrt-o*) in *P. vivax* isolates collected from Kanchanaburi Province, Thailand, from 2022 to 2025. The current frequencies and combinations of key mutations were analyzed without inferring functional or clinical resistance. These findings outline the present distribution of candidate molecular markers and highlight the need for continued surveillance and future hypothesis‑driven functional and clinical studies to support evidence‑based malaria treatment strategies in this region.

## Supplementary Information

Supplementary file 1. Table S1. Nucleotide sequences of primers for the identification of five* Plasmodium* species.

Supplementary file 2. Table S2. Summary of sequencing success and detected mutations across four putative drug resistance genes in 100 *P. vivax* isolates.

Supplementary file 3. Table S3. FASTA sequences of drug‑resistance–associated genes of *P. vivax* isolates analyzed in this study.

Supplementary file 4. Figure S4. Chromatograms of the wild-type *Pvcrt-o* sequence at codon 32 and the *Pvcrt-o* K32T mutant (c.32AAG>ACG).

Supplementary file 5. Table S5. Haplotype and nucleotide diversity of *Pvmdr1*, *Pvdhfr*, *Pvdhps*, and *Pvcrt-o *in *P. vivax* isolates from Kanchanaburi province, Thailand.

## Data Availability

All data supporting the findings of this study are available within the paper and its Supplementary Information.
