# Supplementary material for: Molecular surveillance of Plasmodium vivax drug resistance genes in Kanchanaburi province, Thailand, from 2022 to 2025
Source: Malar J. 2026 Aug 7;25:397. doi: 10.1186/s12936-026-06099-1 (PMC13733103; doi:10.1186/s12936-026-06099-1)
Supplement: Supplementary file 1 [file 12936_2026_6099_MOESM1_ESM.pdf]

Supplementary Data 1

Table S1 Nucleotide sequences of primers for the identification of five *Plasmodium* species

Cycle	Species	Primers name	Sequences (5' - 3')	Amplicon size (bp)	Ref.
Nest 1	*Plasmodium* spp.	rPLU1	TCAAAGATTAAGCCATGCAAGTGA	1,700	Snounou et al., 1993
		rPLU5	CCTGTTGTTGCCTTAAACTTC		
		rPLU6	TTAAAATTGTTGCAGTTAAAACG		
Nest 2	*P. vivax*	rVIV1	CGCTTCTAGCTTAATCCACATAACTGATAC	120	
		rVIV2	ACTTCCAAGCCGAAGCAAAGAAAGTCCTTA		
	P. falciparum	rFAL1	TTAAACTGGTTTGGGAAAACCAAATATATT	205	
		rFAL2	ACACAATGAACTCAATCATGACTACCCGTC		
	P. ovale	rOVA1	ATCTCTTTTGCTATTTTTTAGTATTGGAGA	800	
		rOVA2	GGAAAAGGACACATTAATTGTATCCTAAT		
	P. malariae	rMAL1	ATAACATAGTTGTACGTTAAGAATAACCGC	144	
		rMAL2	AAAATTCCCATGCATAAAAAATTATACAAA		
Nest-2	*P. knowlesi*	Pmk8	GTTAGCGAGAGCCACAAAAAAGCGAAT	153	Imwong et al., 2009
		Pmkr9	ACTCAAAGTAACAAAATCTTCCATA		
