# Supplementary material for: Molecular surveillance of Plasmodium vivax drug resistance genes in Kanchanaburi province, Thailand, from 2022 to 2025
Source: Malar J. 2026 Aug 7;25:397. doi: 10.1186/s12936-026-06099-1 (PMC13733103; doi:10.1186/s12936-026-06099-1)
Supplement: Supplementary file 2 [file 12936_2026_6099_MOESM3_ESM.pdf]

Supplementary Data 3.

Table S3. FASTA sequences of drug-resistance–associated genes of *Plasmodium vivax* isolates analyzed in this study.

**Targeted Genes**	FASTA sequence
Pvmdr1 (604 bp)	
1. Single mutant<br>($\mathbf{M_{958}}$/Y_{976}/L_{1076})	GGATAGTCATGCCCCAGGATTGCTGTCAGCACATATTAACAGAGATGTTCAT<br>TTGTTAAAAACCGGTTTAGTAAATAACATTGTCATTTTTACTCACTTTATAG<br>TGCTCTTCCTTGTGAGT**ATG**GTCATGTCATTTTATTTCTGCCCTATCGTGGC<br>GGCTGTACTGACCGGAACGTACTTCATTTTTATGAGAGTGTTTGCCATCAGA<br>GCGAGGATAGCAGCCAACAAGGATGTAGAGAAGAAGCGAGTCAACCAACCAG<br>GCACAGCATTTGTCTACAACAGTGATGATGAAATATTTAAAGACCCAAGTTT<br>CCTAATTCAGGAGGCATTTTACAATATGAACACGGTCATTATTTACGGGCTG<br>GAGGATTACTTCTGCACACTGATTGAGAAGGCTATTGATTATTCGAATAAAG<br>GACAAAAGAGAAAGACGCTAATAAATTCGATGCTCTGGGGGTTCAGTCAGAG<br>TGCCCAATTTTTCATTAACAGTTTTGCCTACTGGTTTGGTTCCTTCCTAATT<br>AGAAGAGGTACAATACAAGTGGATGACTTTATGAAATCCCTCTTTACCTTTT<br>TATTTACGGGAAGCTACGCCGGGAAGTTGATG
2. Double mutant<br>($\mathbf{M_{958}}$/Y_{976}/$\mathbf{L_{1076}}$)	GGATAGTCATGCCCCAGGATTGCTGTCAGCACATATTAACAGAGATGTTCAT<br>TTGTTAAAAACCGGTTTAGTAAATAACATTGTCATTTTTACTCACTTTATAG<br>TGCTCTTCCTTGTGAGT**ATG**GTCATGTCATTTTATTTCTGCCCTATCGTGGC<br>GGCTGTACTGACCGGAACGTACTTCATTTTTATGAGAGTGTTTGCCATCAGA<br>GCGAGGATAGCAGCCAACAAGGATGTAGAGAAGAAGCGAGTCAACCAACCAG<br>GCACAGCATTTGTCTACAACAGTGATGATGAAATATTTAAAGACCCAAGTTT<br>CCTAATTCAGGAGGCATTTTACAATATGAACACGGTCATTATTTACGGGCTG<br>GAGGATTACTTCTGCACACTGATTGAGAAGGCTATTGATTATTCGAATAAAG<br>GACAAAAGAGAAAGACGCTAATAAATTCGATGCTCTGGGGGTTCAGTCAGAG<br>TGCCCAA**CTT**TTCATTAACAGTTTTGCCTACTGGTTTGGTTCCTTCCTAATT<br>AGAAGAGGTACAATACAAGTGGATGACTTTATGAAATCCCTCTTTACCTTTT<br>TATTTACGGGAAGCTACGCCGGGAAGTTGATG
3. Triple mutant<br>($\mathbf{M_{958}}$/$\mathbf{F_{976}}$/$\mathbf{L_{1076}}$)	GGATAGTCATGCCCCAGGATTGCTGTCAGCACATATTAACAGAGATGTTCAT<br>TTGTTAAAAACCGGTTTAGTAAATAACATTGTCATTTTTACTCACTTTATAG<br>TGCTCTTCCTTGTGAGT**ATG**GTCATGTCATTTTATTTCTGCCCTATCGTGGC<br>GGCTGTACTGACCGGAACG**TTC**TTCATTTTTATGAGAGTGTTTGCCATCAGA<br>GCGAGGATAGCAGCCAACAAGGATGTAGAGAAGAAGCGAGTCAACCAACCAG<br>GCACAGCATTTGTCTACAACAGTGATGATGAAATATTTAAAGACCCAAGTTT<br>CCTAATTCAGGAGGCATTTTACAATATGAACACGGTCATTATTTACGGGCTG<br>GAGGATTACTTCTGCACACTGATTGAGAAGGCTATTGATTATTCGAATAAAG<br>GACAAAAGAGAAAGACGCTAATAAATTCGATGCTCTGGGGGTTCAGTCAGAG<br>TGCCCAA**CTT**TTCATTAACAGTTTTGCCTACTGGTTTGGTTCCTTCCTAATT<br>AGAAGAGGTACAATACAAGTGGATGACTTTATGAAATCCCTCTTTACCTTTT<br>TATTTACGGGAAGCTACGCCGGGAAGTTGATG
Pvdhfr (755 bp)	
1. Wild-Type<br>(F_{57}/S_{58}/T_{61}/S_{117})	CCCCACCACATAACGAAGTAGGAGTAACCGCTTAACCCATGGAGGACCTTTC<br>AGATGTATTTGACATTTACGCCATCTGCGCATGCTGCAAGGTCGCCCCCACC

	AGTGAAGGGACAAAGAATGAACCGTTCAGCCCGCGGACCTTTAGGGGTCTGG<br>GCAATAAGGGGACTCTCCCATGGAAATGCAACTCCGTCGATATGAAGTAC**TT**<br>**CAGC**TCGGTG**ACG**ACCTACGTGGATGAGTCAAAGTACGAGAAGCTAAAGTGG<br>AAGAGGGAGAGGTACCTACGAATGGAAGCCTCACAGGGGGGGGTGACAACA<br>CAAGCGGTGGTGACAACACACACGGTGGTGACAACGCCGACAAGCTGCAAAA<br>CGTCGTGGTCATGGGGAGAAGC**AGC**TGGGAGAGCATCCCCAAGCAGTACAAG<br>CCGCTCCCAAACAGAATCAACGTCGTGCTTTCCAAGACGCTAACAAAGGAAG<br>ACGTGAAGGAAAAGGTCTTCATAATTGACAGCATAGATGACCTACTGCTGCT<br>CTTAAAGAAGCTGAAGTACTACAAATGCTTCATCATTGGGGGAGCACAAGTT<br>TATAGGGAATGCCTAAGTAGAAACTTAATCAAGCAGATCTACTTCACGAGGA<br>TCAACGGCGCCTACCCGTGTGACGTCTTCTTCCCCGAGTTTGACGAAAGCCA<br>GTTTCGGGTGACGTCCGTCAGTGAGGTGTACAACAGCAAGGGCACCACTCTG<br>GACTTTTTGGTTTACAGCAAGGTGGGG
2. Single mutant<br>($\mathbf{L_{57}}$/S_{58}/T_{61}/S_{117})	CCCCACCACATAACGAAGTAGGAGTAACCGCTTAACCCATGGAGGACCTTTC<br>AGATGTATTTGACATTTACGCCATCTGCGCATGCTGCAAGGTCGCCCCCACC<br>AGTGAAGGGACAAAGAATGAACCGTTCAGCCCGCGGACCTTTAGGGGTCTGG<br>GCAATAAGGGGACTCTCCCATGGAAATGCAACTCCGTCGATATGAAGTAC**TT**<br>**G**AGCTCGGTGACGACCTACGTGGATGAGTCAAAGTATGAGAAGCTAAAGTGG<br>AAGAGGGAGAGGTACCTACGAATGGAAGCCTCACAGGGGGGGGTGACAACA<br>CAAGCGGTGGTGACAACACACACGGTGGTGACAACGCCGACAAGCTGCAAAA<br>CGTCGTGGTCATGGGGAGAAGCAGCTGGGAGAGCATCCCCAAGCAGTACAAG<br>CCGCTCCCAAACAGAATCAACGTCGTGCTTTCCAAGACGCTAACAAAGGAAG<br>ACGTGAAGGAAAAGGTCTTCATAATTGACAGCATAGATGACCTACTGCTGCT<br>CTTAAAGAAGCTGAAGTACTACAAATGCTTCATCTTTGGGGGAGCACAAGTT<br>TATAGGGAATGCCTAAGTAGAAACTTAATCAAGCAGATCTACTTCACGAGGA<br>TCAACGGCGCCTACCCGTGTGACGTCTTCTTCCCCGAGTTTGACGAAAGCCA<br>GTTTCGGGTGACGTCCGTCAGTGAGGTGTACAACAGCAAGGGCACCACTCTG<br>GACTTTTTGGTTTACAGCAAGGTGGGG
3. Double mutant<br>(F_{57}/$\mathbf{R_{58}}$/T_{61}/$\mathbf{N_{117}}$)	CCCCACCACATAACGAAGTAGGAGTAACCGCTTAACCCATGGAGGACCTTTC<br>AGATGTATTTGACATTTACGCCATCTGCGCATGCTGCAAGGTCGCCCCCACC<br>AGTGAAGGGACAAAGAATGAACCGTTCAGCCCGCGGACCTTTAGGGGTCTGG<br>GCAATAAGGGGACTCTCCCATGGAAATGCAACTCCGTCGATATGAAGTACTT<br>C**AGG**TCGGTGACGACCTACGTGGATGAGTCAAAGTACGAGAAGCTAAAGTGG<br>AAGAGGGAGAGGTACCTACGAATGGAAGCCTCACAGGGGGGGGTGACAACA<br>CAAGCGGTGGTGACAACACACACGGTGGTGACAACGCCGACAAGCTGCAAAA<br>CGTCGTGGTCATGGGGAGAAGC**AAC**TGGGAGAGCATCCCCAAGCAGTACAAG<br>CCGCTCCCAAACAGAATCAACGTCGTGCTTTCCAAGACGCTAACAAAGGAAG<br>ACGTGAAGGAAAAGGTCTTCATAATTGACAGCATAGATGACCTACTGCTGCT<br>CTTAAAGAAGCTGAAGTACTACAAATGCTTCATCATTGGGGGAGCACAAGTT<br>TATAGGGAATGCCTAAGTAGAAACTTAATCAAGCAGATCTACTTCACGAGGA<br>TCAACGGCGCCTACCCGTGTGACGTCTTCTTCCCCGAGTTTGACGAAAGCCA<br>GTTTCGGGTGACGTCCGTCAGTGAGGTGTACAACAGCAAGGGCACCACTCTG<br>GACTTTTTGGTTTACAGCAAGGTGGGG
4. Quadruple mutant<br>($\mathbf{I_{57}}$/$\mathbf{R_{58}}$/$\mathbf{M_{61}}$/$\mathbf{T_{117}}$)	CCCCACCACATAACGAAGTAGGAGTAACCGCTTAACCCATGGAGGACCTTTC<br>AGATGTATTTGACATTTACGCCATCTGCGCATGCTGCAAGGTCGCCCCCACC<br>AGTGAAGGGACAAAGAATGAACCGTTCAGCCCGCGGACCTTTAGGGGTCTGG<br>GCAATAAGGGGACTCTCCCATGGAAATGCAACTCCGTCGATATGAAGTAC**AT**<br>**AAGG**TCGGTG**ATG**ACCTACGTGGATGAGTCAAAGTATGAGAAGCTAAAGTGG<br>AAGAGGGAGAGGTACCTACGAATGGAAGCCTCACAGGGGGGGGTGACAACA<br>CAAGCGGTGGTGACAACACACACGGTGGTGACAACGCCGACAAGCTGCAAAA<br>CGTCGTGGTCATGGGGAGAAGC**ACC**TGGGAGAGCATCCCCAAGCAGTACAAG

	CCGCTCCCAAACAGAATCAACGTCGTGCTTTCCAAGACGCTAACAAAGGAAG<br>ACGTGAAGGAAAAGGTCTTCATAATTGACAGCATAGATGACCTACTGCTGCT<br>CTTAAAGAAGCTGAAGTACTACAAATGCTTCATCATTGGGGGAGCACAAGTT<br>TATAGGGAATGCCTAAGTAGAAACTTAATCAAGCAGATCTACTTCACGAGGA<br>TCAACGGCGCCTACCCGTGTGACGTCTTCTTCCCCGAGTTTGACGAAAGCCA<br>GTTTCGGGTGACGTCCGTCAGTGAGGTGTACAACAGCAAGGGCACCACTCTG<br>GACTTTTTGGTTTACAGCAAGGTGGGG
5. Quadruple mutant ($\mathbf{L_{57}}$/$\mathbf{R_{58}}$/$\mathbf{M_{61}}$/$\mathbf{T_{117}}$)	CCCCACCACATAACGAAGTAGGAGTAACCGCTTAACCCATGGAGGACCTTTC<br>AGATGTATTTGACATTTACGCCATCTGCGCATGCTGCAAGGTCGCCCCCACC<br>AGTGAAGGGACAAAGAATGAACCGTTCAGCCCGCGGACCTTTAGGGGTCTGG<br>GCAATAAGGGGACTCTCCCATGGAAATGCAACTCCGTCGATATGAAGTAC**TT**<br>**AAGG**TCGGTG**ATG**ACCTACGTGGATGAGTCAAAGTATGAGAAGCTAAAGTGG<br>AAGAGGGAGAGGTACCTACGAATGGAAGCCTCACAGGGGGGGGTGACAACA<br>CAAGCGGTGGTGACAACACACACGGTGGTGACAACGCCGACAAGCTGCAAAA<br>CGTCGTGGTCATGGGGAGAAGC**ACC**TGGGAGAGCATCCCCAAGCAGTACAAG<br>CCGCTCCCAAACAGAATCAACGTCGTGCTTTCCAAGACGCTAACAAAGGAAG<br>ACGTGAAGGAAAAGGTCTTCATAATTGACAGCATAGATGACCTACTGCTGCT<br>CTTAAAGAAGCTGAAGTACTACAAATGCTTCATCATTGGGGGAGCACAAGTT<br>TATAGGGAATGCCTAAGTAGAAACTTAATCAAGCAGATCTACTTCACGAGGA<br>TCAACGGCGCCTACCCGTGTGACGTCTTCTTCCCCGAGTTTGACGAAAGCCA<br>GTTTCGGGTGACGTCCGTCAGTGAGGTGTACAACAGCAAGGGCACCACTCTG<br>GACTTTTTGGTTTACAGCAAGGTGGGG
Pvdhps (731 bp)	
1. Wild Type (A_{383}/K_{512}/A_{553})	GCTGTGGAGAGGATGTTCGAAATGGCAAGTGATGGGGCGAGCGTGATTGACA<br>TCGGGGGGGAATCGTCC**GCC**CCTTATGTGGTCCCCAATCCGAGCGTCACTGA<br>ACGGGATTTGGTCATGCCTGTTTTGAAGCTCTTTAAGGAGGAGTGGCATAAG<br>TTGGAGTGTGAGGTTGGCGGTGGTGCCGTGTGCTGCGCTGCAGCAAGTGATG<br>CCAGGAAAAACGCGCAGAGCTCCCTACAGGGGAAACTACAAAAAGTGAGGGA<br>CGCAAAACCGATCATAAGCATCGACACGGTCAATTATGATCTCTTCAAGGAG<br>TGCGTGGAAGGCGAGTTGGTGGACATCCTAAACGATATCAGCGCGTGCACGC<br>ACAACCCAGAGATTATAAAATTGTTGAGGAGGAAAAACAAGTTCTATAGCGT<br>CGTTTTAATGCACAAGAGGGGAAATCCACACACCATGGAT**AAG**TTAACAAAT<br>TACGATGACCTTATAAGTGACATTAAAAGGTATTTAGAAGATCGGCTACATT<br>TTCTCGTTCTAAATGGGGTACCACGCTACCGAGTTCTCTTTGATGTCGGCCT<br>GGGGTTT**GCC**AAAAAGCACGACCAGTCTATTAAGCTGTTGCAGCATATTCAC<br>GTTTACGATGAGTACCCGCTGTTTCTTGGCTACTCGAGGAAGCGCTTTATTG<br>TCCACTGCATGGGGAAGGGTGGCGCGGCCATCGGGGAGTGCAGACTGATGAG<br>CGG
2. Single mutant ($\mathbf{G_{383}}$/K_{512}/A_{553})	GCTGTGGAGAGGATGTTCGAAATGGCAAGTGATGGGGCGAGCGTGATTGACA<br>TCGGGGGGGAATCGTCC**GGC**CCTTATGTGGTCCCCAATCCGAGCGTCACTGA<br>ACGGGATTTGGTCATGCCTGTTTTGAAGCTCTTTAAGGAGGAGTGGCATAAG<br>TTGGAGTGTGAGGTTGGCGGTGGTGCCGTGTGCTGCGCTGCAGCAAGTGATG<br>CCAGGAAAAACGCGCAGAGCTCCCTACAGGGGAAACTACAAAAAGTGAGGGA<br>CGCAAAACCGATCATAAGCATCGACACGGTCAATTATGATCTCTTCAAGGAG<br>TGCGTGGAAGGCGAGTTGGTGGACATCCTAAACGATATCAGCGCGTGCACGC<br>ACAACCCAGAGATTATAAAATTGTTGAGGAGGAAAAACAAGTTCTATAGCGT<br>CGTTTTAATGCACAAGAGGGGAAATCCACACACCATGGATAAGTTAACAAAT<br>TACGATGACCTTATAAGTGACATTAAAAGGTATTTAGAAGATCGGCTACATT<br>TTCTCGTTCTAAATGGGGTACCACGCTACCGAGTTCTCTTTGATGTCGGCCT

	GGGGTTTGCCAAAAAGCACGACCAGTCTATTAAGCTGTTGCAGCATATTCAC<br>GTTTACGATGAGTACCCGCTGTTTCTTGGCTACTCGAGGAAGCGCTTTATTG<br>TCCACTGCATGGGGAAGGGTGGCGCGGCCATCGGGGAGTGCAGACTGATGAG<br>CGG
3. Double mutant<br>($\mathbf{G_{383}}$/K_{512}/$\mathbf{G_{553}}$)	GCTGTGGAGAGGATGTTCGAAATGGCAAGTGATGGGGCGAGCGTGATTGACA<br>TCGGGGGGGAATCGTCC**GGC**CCTTATGTGGTCCCCAATCCGAGCGTCACTGA<br>ACGGGATTTGGTCATGCCTGTTTTGAAGCTCTTTAAGGAGGAGTGGCATAAG<br>TTGGAGTGTGAGGTTGGCGGTGGTGCCGTGTGCTGCGCTGCAGCAAGTGATG<br>CCAGGAAAAACGCGCAGAGCTCCCTACAGGGGAAACTACAAAAAGTGAGGGA<br>CGCAAAACCGATCATAAGCATCGACACGGTCAATTATGATCTCTTCAAGGAG<br>TGCGTGGAAGGCGAGTTGGTGGACATCCTAAACGATATCAGCGCGTGCACGC<br>ACAACCCAGAGATTATAAAATTGTTGAGGAGGAAAAACAAGTTCTATAGCGT<br>CGTTTTAATGCACAAGAGGGGAAATCCACACACCATGGATAAGTTAACAAAT<br>TACGATGACCTTATAAGTGACATTAAAAGGTATTTAGAAGATCGGCTACATT<br>TTCTCGTTCTAAATGGGGTACCACGCTACCGAGTTCTCTTTGATGTCGGCCT<br>GGGGTTT**GGC**AAAAAGCACGACCAGTCTATTAAGCTGTTGCAGCATATTCAC<br>GTTTACGATGAGTACCCGCTGTTTCTTGGCTACTCGAGGAAGCGCTTTATTG<br>TCCACTGCATGGGGAAGGGTGGCGCGGCCATCGGGGAGTGCAGACTGATGAG<br>CGG
4. Triple mutant<br>($\mathbf{G_{383}}$/$\mathbf{M_{512}}$/$\mathbf{G_{553}}$)	GCTGTGGAGAGGATGTTCGAAATGGCAAGTGATGGGGCGAGCGTGATTGACA<br>TCGGGGGGGAATCGTCC**GGC**CCTTATGTGGTCCCCAATCCGAGCGTCACTGA<br>ACGGGATTTGGTCATGCCTGTTTTGAAGCTCTTTAAGGAGGAGTGGCATAAG<br>TTGGAGTGTGAGGTTGGCGGTGGTGCCGTGTGCTGCGCTGCAGCAAGTGATG<br>CCAGGAAAAACGCGCAGAGCTCCCTACAGGGGAAACTACAAAAAGTGAGGGA<br>CGCAAAACCGATCATAAGCATCGACACGGTCAATTATGATCTCTTCAAGGAG<br>TGCGTGGAAGGCGAGTTGGTGGACATCCTAAACGATATCAGCGCGTGCACGC<br>ACAACCCAGAGATTATAAAATTGTTGAGGAGGAAAAACAAGTTCTATAGCGT<br>CGTTTTAATGCACAAGAGGGGAAATCCACACACCATGGAT**ATG**TTAACAAAT<br>TACGATGACCTTATAAGTGACATTAAAAGGTATTTAGAAGATCGGCTACATT<br>TTCTCGTTCTAAATGGGGTACCACGCTACCGAGTTCTCTTTGATGTCGGCCT<br>GGGGTTT**GGC**AAAAAGCACGACCAGTCTATTAAGCTGTTGCAGCATATTCAC<br>GTTTACGATGAGTACCCGCTGTTTCTTGGCTACTCGAGGAAGCGCTTTATTG<br>TCCACTGCATGGGGAAGGGTGGCGCGGCCATCGGGGAGTGCAGACTGATGAG<br>CGG
Pvcrt-o (1186 bp)	
1. Wild Type	AAGAGCCGTCTAGCCATCCGCGTCACGTTATCTGCATCACGTTATCTGCATC<br>CCGTCATCCGCCTCACTCCATCTGCGCCACCGCTACCCCTAACGCACAATGA<br>CCATCCTGAAAAAGAAGAAGAAGGGGAGTCCCCAAATAACCCCCGATGAACG<br>TTACCGGGAGTTGGATAGCCACGCACAGAATGAGAGT**AAG**GCAGGCCCTTAG<br>CGAAGGGTCCCCCTGACCACCGTGCAGGAGAGTGGCGAGACAACCCGCTGCC<br>CCTACCCGCGAGGGGTGTTTTACCCTTTACGTGAGGCACTCCATTGATCAAC<br>CCCCCTAATCACTTCTCTAATAAACAACTGCTTTCCCCCCACAGGCGAAATA<br>CAAGAGGACGTCCCCATCAGCAGAAAAATCGCCAATTTTTTGAAGCTAGCTT<br>ATAATGAAATTCGGGAAAATATTTCCATTTATCTGCTGATTATCGTTTACCT<br>GTGCGTATGTGTAATGAATAAACTGCTGGCAAAGAGGACTCTGAAGAAGATT<br>GGGAACTACAGCTTTGTAACCTCCGAGACGCACAACTGCATATGCATGGTCG<br>TTTTCTTTGCCTTGTACTTTATGTTTGGTCGCCGGGTCATGTCCGCCAAGGT<br>GAGCAGCGCAGCAGATGGGTTATTCTTTAATAACTACACCATCTAATCGCTC<br>CTTCTGCATGCGTTATTGGAAGGGGGGAGAGCATCCTGCGGAGAGGCAGACA

	TATCCCCGCAGAGTGCGTTCCACTGGTGGTGGTGCCCCCAGTGCATCCATCG<br>GGAGTACCTCCTTCTATATACCCCTTCGCCTTTTATATATACCCCTTTACCG<br>CTTTACCTTCCCCCCGCAGGAGAGACACCGAAACTTCGGAGTTCAATTTTTG<br>CTAATCTCGCTGCTGGACGCCTGCTCAGTTATCATCGCGTTTATAGGTAATT<br>CGCGCGGCGAAGCGGCGGCTCGCCCGGTAGAAGCCGCGTCGATTTTAACCCC<br>TCATCTGCTTAACCTCTCCTTTACTTCACTTCCCTCCTTTTCGCGCGTCTTT<br>TCCCCCCCGTCAGGCCTCACAAGAACCACCGGGAACATCCAGTCCTTCGTCA<br>TGCAGCTGAGCATTCCGATAAACATGTTTTTCTGCTTTTTGATTTTGCGATA<br>CAGGTAGGGGAGAGGCGGCATAATCGGGTGTAGAGGGAAACTC
2. Single mutant<br>(**K10 insertion**/K$_{32}$)	AAGAGCCGTCTAGCCATCCGCGTCACGTTATCTGCATCACGTTATCTGCATC<br>CCGTCATCCGCCTCACTCCATCTGCGCCACCGCTACCCCTAACGCACAATGA<br>CCATCCTGAAAAAGAAGAAGAAG**AAG**GGGAGTCCCCAAATAACCCCCGATGA<br>ACGTTACCGGGAGTTGGATAGCCACGCACAGAATGAGAGT**AAG**GCAGGCCCT<br>TAGCGAAGGGTCCCCCTGACCACCGTGCAGGAGAGTGGCGAGACAACCCGCT<br>GCCCCTACCCGCGAGGGGTGTTTTACCCTTTACGTGAGGCACTCCATTGATC<br>AACCCCCCTAATCACTTCTCTAATAAACAACTGCTTTCCCCCCACAGGCGAA<br>ATACAAGAGGACGTCCCCATCAGCAGAAAAATCGCCAATTTTTTGAAGCTAG<br>CTTATAATGAAATTCGGGAAAATATTTCCATTTATCTGCTGATTATCGTTTA<br>CCTGTGCGTATGTGTAATGAATAAACTGCTGGCAAAGAGGACTCTGAAGAAG<br>ATTGGGAACTACAGCTTTGTAACCTCCGAGACGCACAACTGCATATGCATGG<br>TCGTTTTCTTTGCCTTGTACTTTATGTTTGGTCGCCGGGTCATGTCCGCCAA<br>GGTGAGCAGCGCAGCAGATGGGTTATTCTTTAATAACTACACCATCTAATCG<br>CTCCTTCTGCATGCGTTATTGGAAGGGGGGAGAGCATCCTGCGGAGAGGCAG<br>ACATATCCCCGCAGAGTGCGTTCCACTGGTGGTGGTGCCCCCAGTGCATCCA<br>TCGGGAGTACCTCCTTCTATATACCCCTTCGCCTTTTATATATACCCCTTTA<br>CCGCTTTACCTTCCCCCCGCAGGAGAGACACCGAAACTTCGGAGTTCAATTT<br>TTGCTAATCTCGCTGCTGGACGCCTGCTCAGTTATCATCGCGTTTATAGGTA<br>ATTCGCGCGGCGAAGCGGCGGCTCGCCCGGTAGAAGCCGCGTCGATTTTAAC<br>CCCTCATCTGCTTAACCTCTCCTTTACTTCACTTCCCTCCTTTTCGCGCGTC<br>TTTTCCCCCCCGTCAGGCCTCACAAGAACCACCGGGAACATCCAGTCCTTCG<br>TCATGCAGCTGAGCATTCCGATAAACATGTTTTTCTGCTTTTTGATTTTGCG<br>ATACAGGTAGGGGAGAGGCGGCATAATCGGGTGTAGAGGGAAACTC
3. Single mutant<br>(K$_{32}$ mutation/**T$_{32}$**)	AAGAGCCGTCTAGCCATCCGCGTCACGTTATCTGCATCACGTTATCTGCATC<br>CCGTCATCCGCCTCACTCCATCTGCGCCACCGCTACCCCTAACGCACAATGA<br>CCATCCTGAAAAAGAAGAAGAAGGGGAGTCCCCAAATAACCCCCGATGAACG<br>TTACCGGGAGTTGGATAGCCACGCACAGAATGAGAGT**ACG**GCAGGCCCTTAG<br>CGAAGGGTCCCCCTGACCACCGTGCAGGAGAGTGGCGAGACAACCCGCTGCC<br>CCTACCCGCGAGGGGTGTTTTACCCTTTACGTGAGGCACTCCATTGATCAAC<br>CCCCCTAATCACTTCTCTAATAAACAACTGCTTTCCCCCCACAGGCGAAATA<br>CAAGAGGACGTCCCCATCAGCAGAAAAATCGCCAATTTTTTGAAGCTAGCTT<br>ATAATGAAATTCGGGAAAATATTTCCATTTATCTGCTGATTATCGTTTACCT<br>GTGCGTATGTGTAATGAATAAACTGCTGGCAAAGAGGACTCTGAAGAAGATT<br>GGGAACTACAGCTTTGTAACCTCCGAGACGCACAACTGCATATGCATGGTCG<br>TTTTCTTTGCCTTGTACTTTATGTTTGGTCGCCGGGTCATGTCCGCCAAGGT<br>GAGCAGCGCAGCAGATGGGTTATTCTTTAATAACTACACCATCTAATCGCTC<br>CTTCTGCATGCGTTATTGGAAGGGGGGAGAGCATCCTGCGGAGAGGCAGACA<br>TATCCCCGCAGAGTGCGTTCCACTGGTGGTGGTGCCCCCAGTGCATCCATCG<br>GGAGTACCTCCTTCTATATACCCCTTCGCCTTTTATATATACCCCTTTACCG<br>CTTTACCTTCCCCCCGCAGGAGAGACACCGAAACTTCGGAGTTCAATTTTTG<br>CTAATCTCGCTGCTGGACGCCTGCTCAGTTATCATCGCGTTTATAGGTAATT<br>CGCGCGGCGAAGCGGCGGCTCGCCCGGTAGAAGCCGCGTCGATTTTAACCCC

	TCATCTGCTTAACCTCTCCTTTACTTCACTTCCCTCCTTTTCGCGCGTCTTT<br>TCCCCCCCGTCAGGCCTCACAAGAACCACCGGGAACATCCAGTCCTTCGTCA<br>TGCAGCTGAGCATTCCGATAAACATGTTTTTCTGCTTTTTGATTTTGCGATA<br>CAGGTAGGGGAGAGGCGGCATAATCGGGTGTAGAGGGAAACTC
