# Supplementary material for: Molecular surveillance of Plasmodium vivax drug resistance genes in Kanchanaburi province, Thailand, from 2022 to 2025
Source: Malar J. 2026 Aug 7;25:397. doi: 10.1186/s12936-026-06099-1 (PMC13733103; doi:10.1186/s12936-026-06099-1)

Supplementary Data 4

Figure S4. Chromatograms of the wild-type *Pvcrt-o* sequence at codon 32 and the *Pvcrt-o* K32T mutant (c.32AAG>ACG).

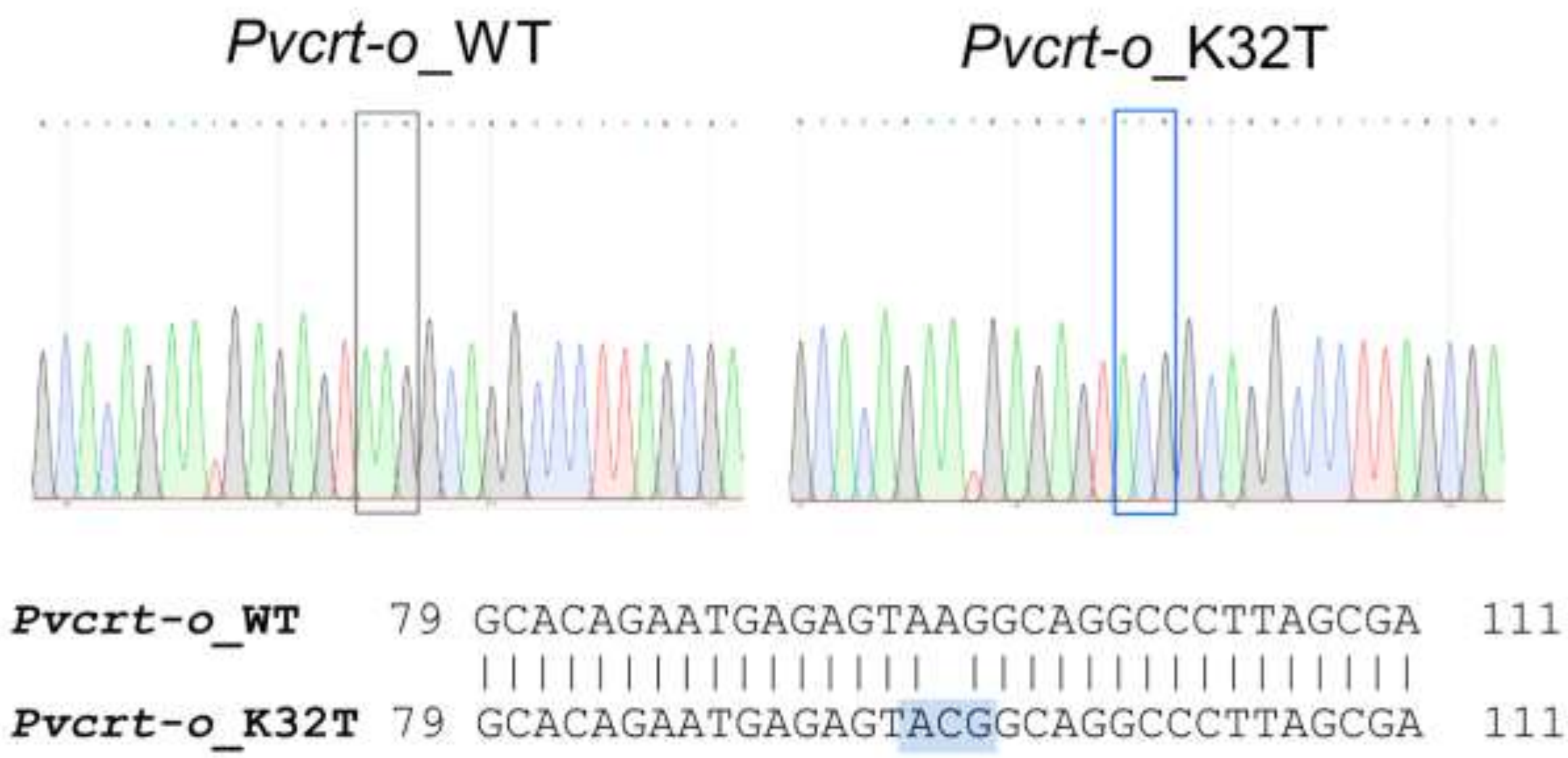

Supplement: Supplementary file 3 [file 12936_2026_6099_MOESM4_ESM.pdf]
