# Supplementary material for: Molecular surveillance of Plasmodium vivax drug resistance genes in Kanchanaburi province, Thailand, from 2022 to 2025
Source: Malar J. 2026 Aug 7;25:397. doi: 10.1186/s12936-026-06099-1 (PMC13733103; doi:10.1186/s12936-026-06099-1)
Supplement: Supplementary file 4 [file 12936_2026_6099_MOESM5_ESM.pdf]

Supplementary Data 5

Table S5. Haplotype and nucleotide diversity of *Pvmdr1*, *Pvdhfr*, *Pvdhps*, and *Pvcrt-o* in *P. vivax* isolates from Kanchanaburi province, Thailand.

**Gene**	**n**	**Haplotypes**	**Haplotype diversity (Hd) ± Standard Deviation (SD)**	**π**
Pvmdr1	99	3	0.5688 ± 0.025	0.00139
Pvdhfr	99	5	0.6785 ± 0.030	0.00287
Pvdhps	98	4	0.6424 ± 0.031	0.00234
Pvcrt-o	77	3	0.6550 ± 0.034	0.00217
